# Supplementary material for: HIVprotI: an integrated web based platform for prediction and design of HIV proteins inhibitors
Source: J Cheminform. 2018 Mar 9;10:12. doi: 10.1186/s13321-018-0266-y (PMC5845081; doi:10.1186/s13321-018-0266-y)
Supplement: Supplementary file 2 — Additional file 2. Source code of HIVProtI web server. [file 13321_2018_266_MOESM2_ESM.zip › HIVprotI_Source-code/hivprotI/weka-3-6-6/doc/weka/core/SpecialFunctions.html]

SpecialFunctions


---


|  |  |  |
| --- | --- | --- |
| **Overview** \| **Package** \| **Class** \| **Tree** \| **Deprecated** \| **Index** \| **Help** \| **Weka's home** | |  |
| **PREV CLASS**   **NEXT CLASS** | **FRAMES**    **NO FRAMES**     **All Classes** |  |
| SUMMARY: NESTED \| FIELD \| CONSTR \| METHOD | DETAIL: FIELD \| CONSTR \| METHOD |  |


---


## weka.core Class SpecialFunctions

```
java.lang.Object
  weka.core.SpecialFunctions
```

**All Implemented Interfaces:**: RevisionHandler

---

``` public final class SpecialFunctions extends java.lang.Object implements RevisionHandler ```

Class implementing some mathematical functions.

**Version:**
:   $Revision: 1.8 $

**Author:**
:   Eibe Frank (eibe@cs.waikato.ac.nz)

---

**Constructor Summary**

`SpecialFunctions()`


| **Method Summary** | |
| --- | --- |
| `java.lang.String` | `getRevision()`             Returns the revision string. |
| `static double` | `lnFactorial(double x)`             Returns natural logarithm of factorial using gamma function. |
| `static double` | `log2Binomial(double a, double b)`             Returns base 2 logarithm of binomial coefficient using gamma function. |
| `static double` | `log2Multinomial(double a, double[] bs)`             Returns base 2 logarithm of multinomial using gamma function. |
| `static void` | `main(java.lang.String[] ops)`             Main method for testing this class. |

**Methods inherited from class java.lang.Object**

`equals, getClass, hashCode, notify, notifyAll, toString, wait, wait, wait`

**Constructor Detail**

### SpecialFunctions

```
public SpecialFunctions()
```


**Method Detail**

### lnFactorial

```
public static double lnFactorial(double x)
```

:   Returns natural logarithm of factorial using gamma function.

    :   **Parameters:**: `x` - the value **Returns:**: natural logarithm of factorial

---


### log2Binomial

```
public static double log2Binomial(double a,
                                  double b)
```

:   Returns base 2 logarithm of binomial coefficient using gamma function.

    :   **Parameters:**: `a` - upper part of binomial coefficient: `b` - lower part **Returns:**: the base 2 logarithm of the binominal coefficient a over b

---


### log2Multinomial

```
public static double log2Multinomial(double a,
                                     double[] bs)
```

:   Returns base 2 logarithm of multinomial using gamma function.

    :   **Parameters:**: `a` - upper part of multinomial coefficient: `bs` - lower part **Returns:**: multinomial coefficient of a over the bs

---


### getRevision

```
public java.lang.String getRevision()
```

:   Returns the revision string.

    :   **Specified by:**: `getRevision` in interface `RevisionHandler`
    :   **Returns:**: the revision

---


### main

```
public static void main(java.lang.String[] ops)
```

:   Main method for testing this class.


---


|  |  |  |
| --- | --- | --- |
| **Overview** \| **Package** \| **Class** \| **Tree** \| **Deprecated** \| **Index** \| **Help** \| **Weka's home** | |  |
| **PREV CLASS**   **NEXT CLASS** | **FRAMES**    **NO FRAMES**     **All Classes** |  |
| SUMMARY: NESTED \| FIELD \| CONSTR \| METHOD | DETAIL: FIELD \| CONSTR \| METHOD |  |


---
